# Spirituality and Health Summer Internship Program: Adapting Clinical Pastoral Education for Medical Student Instruction in Patient Spirituality

**DOI:** 10.1089/pmr.2024.0101

**Published:** 2025-04-22

**Authors:** Nasser Douge, Rhoda Toperzer, Horace M. DeLisser

**Affiliations:** ^1^ Academic Programs Office, Perelman School of Medicine, University of Pennsylvania, Philadelphia, Pennsylvania, USA.; ^2^ Pulmonary, Allergy and Critical Care Division, Department of Medicine, Perelman School of Medicine, University of Pennsylvania, Philadelphia, Pennsylvania, USA.

**Keywords:** medical education, patient spirituality, spiritual care, spirituality and health

## Abstract

**Background:**

Training in spirituality and spiritual care is limited in medical education. A potentially novel approach for addressing these gaps in medical training is an immersive, experiential internship focused on patient spirituality and spiritual care based on pedagogical approaches adapted from clinical pastoral education (CPE).

**Methods:**

Mixed method analyses were undertaken of participants pre- and post-program surveys and comments to assess the first five years of the six-week Spirituality And Health Summer Internship Program, modeled on a unit of CPE, for first-year medical students.

**Results:**

On a 5-point Likert scale (1 = poor/strongly disagree, 5 = excellent/strongly agree) participants rated the educational value (4.7, standard deviation [SD] = 0.3) and overall quality (4.4, SD = 0.35) of the internship highly and strongly endorsed they would recommend the internship to peers (4.48, SD = 0.36). Participants strongly valued (4.58, SD = 0.35) the opportunity to visit and have conversations with patients as a core activity of the internship. Following the internship, participants reported significant (*p* = 0.013 to *p* < 0.0001) increases in their (1) awareness of how spirituality influences their lives, (2) knowledge of the potential impact of spirituality on the patient experience, and (3) knowledge of the role of spirituality in the lives of health care providers. Significant increases were also noted in participants’ comfort in (1) talking to patients, (2) talking about spirituality, and (3) talking to patients about spirituality.

**Conclusions:**

A medical student summer internship focused on patient spirituality and spiritual care modeled after CPE provides a level of immersion in this content not obtainable in typical medical school curricula.

## Introduction 

Spiritual care has increasingly been recognized as a vital component of medical care, with growing evidence of the impact of patient spirituality on patients’ mental and physical health, lifestyle decisions, and all-cause mortality.^1–3^ Notwithstanding trends toward increased secularization and nonreligious affiliation in the United States,^4,5^ patients remain open to provider efforts to engage their spirituality,^6–9^ with patients and family reporting that this aspect of care is often inadequately addressed.^10–13^ Further, although spiritual care is a core element of quality palliative care,^14,15^ there are gaps in the provision of this care related to inadequate assessment, insufficient clinician training, sub-optimal use of chaplains and poor integration into the plan of care^16,17^ Despite the significant role of spirituality inpatient experiences, and unmet patient desires for spiritual care, training in spirituality and spiritual care is limited in medical education.^18–20^ These deficiencies have prompted efforts to enhance the teaching of spirituality and health during medical training, with calls for new approaches for providing this instruction.^1,2,18–20^

Clinical pastoral education (CPE) provides professional training in spiritual care for individuals seeking professional spiritual care certification or to further deepen their spiritual ministry.^21–23^ This training is typically provided as single units of 10–12 weeks or as a year-long experience composed of 3 or 4 consecutive units at sites accredited by the Association for Clinical Pastoral Education, Inc.^23^ While the content varies according to the training site, CPE emphasizes building on previous knowledge and skills, self-reflection and personal growth in the context of the trainee’s belief system, development of communication and relational skills, and fostering competence in engaging and supporting patients/families in crisis, spiritual, existential or otherwise.^23–25^ Further, CPE has been adapted for other health care professionals, with participants demonstrating increased self-awareness, more empathic communication, and enhanced leadership skills.^26,27^ Given these features, CPE potentially provides a model for teaching spirituality and spiritual care to spiritually diverse medical students that concurrently promotes the development of humanistic physician skills. In this report, we describe the initial outcomes of a summer internship, modeled on a unit of CPE, for first-year medical students that provides an immersion experience in spirituality and health.

## Methods

### Program description

Established in 2016, the Spirituality and Health Summer Internship Program (SH-SIP) provides an immersion educational experience for medical students between their first and second years at the Perelman School of Medicine that promotes professional development and clinically relevant skills and knowledge pertinent to physician and patient spirituality. Programs are presented in Table 1.

**Table 1.** Instructional Goals of the Spirituality and Health Summer Internship

Goal	Description
1	Examine their personal experiences, attitudes and beliefs regarding religion/spirituality
2	Develop approaches for processing their reactions to loss and grief
3	Gain knowledge of how attitudes and beliefs about religion/spirituality impact physician behavior
4	Experience the spiritual resources and concerns of patients and their families
5	Develop skills for eliciting and responding to the spiritual/religious resources and concerns of patients
6	Be exposed to research regarding spirituality and health
7	Learn about major spiritual/religious traditions
8	Learn how physicians integrate their spiritual/religious values into medical practice
9	Appreciate the interdisciplinary dimensions of spiritual care as provided by physicians, nurses, social workers and spiritual providers (chaplains, community clergy)
10	Learn about community agencies and programs whose religious/spiritual values are enlisted in the provision of care

This full-time, six-week program, for which students receive a stipend of $3600, is taught by a board-certified pastoral care educator. The program involves eight core instructional elements (Table 2): a capstone presentation, individual narrative presentations, individual one-on-one individual meetings with the program facilitator/instructor, interfaith experiences, interpersonal group conversations, mindfulness/meditation training, patient/family visitations, and visitation presentations. These core elements are supplemented by didactic presentations on pertinent topics, a relational communication skills workshop, journal club and clinical case discussions, trauma chaplain shadowing, and interviews of physicians about the integration of spirituality into their careers.

**Table 2.** Core Instructional Elements

No.	Core element	Comment
1	Capstone presentation	At the end of the program, students complete an individual capstone project for the course through which students reflect on and explore one or more of the themes of the program. Each student then gives a 20–25-minute presentation on project to the group in a way that is engaging for their peers and provocative of discussion.
2	Individual narrative presentations	Over the course of the program, each student formally shares their personal story.
3	Individual sessions	Students meet at least three times over the course of the program with the program facilitator/instructor to discuss individual internship experiences in a confidential setting.
4	Interfaith experiences	There are opportunities to have an extended visit to a religious community or faith-based program, the nature of which varied from year to year.
5	Interpersonal group conversations	These sessions provide an opportunity to discuss group theory and explore their awareness and experience of groups.
6	Mindfulness meditation training	Training is provided weekly for learning and practicing mindfulness meditation.
7	Patient/family visitations	Students visit and engage patients and families for 12–15 hours a week, with the goal of following them over consecutive days and weeks. The number of patients seen, and the length of time spent with each patient, are left for the student to decide. Students write about these patient experiences and share them with their peers.
8	Visitation presentations	Written verbatims of patient encounters are presented to the peer group. These discussions explore communication techniques, the feelings and responses evoked, and the nature of the dynamic relationship between the student and the patient.

### Program evaluation

#### Data acquisition

Participants anonymously completed pre- and post-program surveys (see Supplementary Data) that included a 5-point Likert-style prompts to determine self-assessments of their awareness and knowledge of spirituality and their comfort in engaging patients around spirituality/religion. In the pre-survey students were asked to describe three things they hope to learn from the internship, while in the post-survey they were asked to indicate three things they learned from the internship. The post-program survey also had questions using a 5-point Likert scale (1, poor/strongly disagree; 2, fair/disagree; 3, good/neutral; 4, very good/agree; 5, excellent), with free text comments, to assess the overall educational effectiveness of the internship and students’ ratings of the core program elements. Participants were informed that the data would be collected anonymously and used for program evaluation and improvement.

#### Data analysis

Each Likert response was assigned a numerical value from 1 to 5 and results were analyzed using calculations of means and standard deviations (SD) using Microsoft Excel. Percentages were calculated using the same program for gender, ethnicity, and participation by year. Statistical significance was determined using a two-tailed *t* test. Two authors (N.D. and H.M.D.) independently analyzed all free text responses using a thematic analysis involving qualitative content analysis with inductive reasoning.^28^ Themes were delineated with specific qualitative examples from survey responses ensuring each theme had sufficient primary data as supporting evidence. Software was not used for qualitative analysis. This study was performed in accordance with the Institutional Review Board of the University of Pennsylvania and was deemed to be exempt.

## Results

### Student demographics

There were 28 participants in the SH-SIP from the years 2016 to 2021: 5 in 2016, 5 in 2017, 7 in 2018, 7 in 2019, and 4 in 2021 (Table 3). The program was paused in 2020 due to COVID. Of the participants, 71% were female and 29% were male. Students were ethnically diverse with 39% identifying as White, 32% as Asian, 11% as Black, and 18% as Hispanic. Of the 28 participants, 24 responded to the surveys.

**Table 3.** Demographics: Total Enrollment (*n* = 28)

Demographic	Number	Percent of total
Gender (self-identified)		
Male	8	28.6
Female	20	71.4
Race/ethnicity (self-identified)		
White	11	39.3
Asian	9	32.1
Black/African American	3	10.7
Hispanic/Latino/Latinx	5	17.9
Year breakdown		
2016	5	17.9
2017	5	17.9
2018	7	25.0
2019	7	25.0
2021	4	14.2

### Students’ overall evaluation of the program

Students were queried at the end of the program about their sense of the program’s educational effectiveness and value (Table 4). Using a 5-point Likert scale (1 = poor/strongly disagree, 5 excellent/strongly agree) students’ ratings of the educational value and overall quality of the program were high, respectively, 4.7 (SD 0.3) and 4.4 (SD 0.35), with respondents scoring the statement, “I would recommend this program to my peers” at 4.48 (SD = 0.36). These data indicate the SH-SIP was well received and were supported by post-survey comments:

**Table 4.** Overall Program Evaluation

No.	Survey item	2016 (*n* = 5)	2017 (*n* = 5)	2018 (*n* = 5)	2019 (*n* = 6)	2021 (*n* = 2)	Mean (SD)
1	Clarity of program goals	3.2	3.2	3.4	3.5	3.5	3.36 (0.15)
2	How well the program achieves stated goals	4.2	3.8	3.6	3.7	3.5	3.76 (0.27)
3	Educational value of the program	4.8	4.8	4.2	4.7	5	4.7 (0.30)
4	Overall rating/quality of program	4.4	4.2	4.2	4.2	5	4.4 (0.35)
5	I would recommend this program to other students	4.8	4.2	4.2	4.2	5	4.48 (0.39)

SD, standard deviation.

Overall, the program was excellent. I enjoyed the quality of the information I got from it and found it extremely valuable.

Thank you for such an unexpectedly reflective, and thought-provoking, [mind]-expanding summer. I loved our time together.

Ratings of the clarity of program’s goals (3.36, SD = 0.15) and how well those goals were achieved (3.76, SD = 0.84) were rated as good to very good.

### Student assessments of the core elements of the internship

Program activities were individually rated by participants on a 5-point Likert scale (1 = poor, 5 = excellent) following the internship (Table 5). Students strongly valued the opportunity to visit patients, rating this activity at 4.58 (SD = 0.35). This is reflected in post-survey comments regarding the humanistic insights students gained from their patient visitations about the patient experience:

**Table 5.** Evaluations of Core Program Elements

No.	Survey item	2016 (*n* = 5)	2017 (*n* = 5)	2018 (*n* = 5)	2019 (*n* = 6)	2021 (*n* = 2)	Means (SD)
1	Capstone presentations	4.2	4.8	4.4	4.5	5	4.58 (0.32)
2	Individual narrative (life stories) presentations	ND	4.6	4	4.7	ND	4.43 (0.38)
3	Individual sessions	3.8	4.2	3.4	3.8	4	3.84 (0.30)
4	Interfaith experiences	4.44	4.34	3.58	3.5	4.89	4.15 (0.59)
5	Interpersonal group conversations	3.4	3.4	2.8	2	5	3.32 (1.10)
6	Mindfulness meditation	4.8	4.8	3	3.5	5	4.22 (0.91)
7	Patient visitations	4	4.8	4.8	4.8	4.5	4.58 (0.35)
8	Visitation presentations	ND	3.8	3.2	3.2	5	3.80 (0.84)

ND, No Data.

This internship gave me a great appreciation for the value of simply being with patients. There is clearly value in using our medical knowledge to do things for the patient, but that is insufficient to truly care for and contribute to the healing of the patient. Medicine is more than knowledge and technical skill; it is an art form.

[I learned] patterns in the existential reflections patients shared in the midst of suffering and in proximity to death: I noticed many similar themes across very sick patients regarding where they find their strength, how they make sense of their situation, and their views on the afterlife.

Additionally, students highly valued the interfaith experiences, rating these events at 4.15 (SD = 0.59).

Our religious immersion experiences highlighted for me the importance of not only reading about difference but truly exposing myself to it. Reading about Islam and watching YouTube videos gave me a good starting point, but it wasn’t until our immersion experience that I was able to begin considering Islam from the framework and perspective of those who practice it.

Our site visits to all of the religious institutions/services gave me an immense appreciation for the richness of their traditions and the conviction with which they hold their faith. Interacting with these traditions and philosophies was also very humbling in the way it made me realize how many important values are shared across religions.

Other core program elements that were also rated highly were the capstone presentations (4.58, SD = 0.32), the individual narrative sessions (4.43, SD = 0.38), and the mindfulness meditation (4.22, SD = 0.91). Rating them somewhat lower (although still in the good to very good range), respondents scored the individual sessions at 3.84 (SD = 0.30), the visitation presentations at 3.80 (SD = 0.84), and the interpersonal group conversations at 3.32 (SD = 1.10).

### Students’ self-assessments of their learning

The responses of participants to the question, *What would you like to learn this summer*, landed most frequently in two broad categories. There was first a desire to develop an “awareness of spirituality and religion in my own life and the awareness of how these beliefs influence my life and my choices.” In addition, participants wanted to be able to “better communicate with patients (bedside manner, pacing, what to say when the patient is very quiet)” as well as “relate to any patient regardless of their background, (finding) that common ground that shows that I as a health care provider care about them as an individual.” As described below, the students’ assessments of their learning indicated these expectations were met.

#### Awareness and knowledge about spirituality

Students were queried on a 5-point Likert scale (1 = poor, 5 = excellent) before and after the summer internship on their knowledge and awareness of spirituality related to themselves, patient care, and the lives of health care providers (Table 6, Section A). After the summer internship, students reported a significant increase in their awareness of how spirituality influences their life (*p* = 0.013), their knowledge of the potential impact of spirituality on the patient experience (*p* < 0.001), and their knowledge of the role of spirituality in the lives of health care providers (*p* < 0.001). These data are in line with post-program student comments indicating they had a deeper and richer understanding of how to integrate spirituality into their careers as providers. Responses to the question*, What were the three most important things you learned this summer*, included:

**Table 6.** Student Self-Evaluations of Their Knowledge of Spirituality and Comfort with Engaging Patients Around Spirituality

	Survey	Pre					Post					
	Survey items	2017 (*n* = 5)	2018 (*n* = 6)	2019 (*n* = 7)	2021 (*n* = 4)	Mean (SD)	2017 (*n* = 5)	2018 (*n* = 5)	2019 (*n* = 6)	2021 (*n* = 3)	Mean (SD)	*p-*Value
	Section A											
1	The degree of your awareness of your spirituality and its impact or influence on your life	2.8	4	2.9	3	3.175 (0.56)	4.4	4.4	3.8	4.67	4.32 (0.37)	0.013
2	The degree of your knowledge of the potential impact or influence of spirituality on the patient experience	2.2	2.8	2	3	2.5 (0.48)	4.4	4	4.3	4.67	4.34 (0.27)	<0.0001
3	The degree of your knowledge of the potential role of spirituality in the lives (personal and professional) of health care providers	2	2.5	1.6	3	2.275 (0.61)	3.6	3.2	4.2	4.67	3.92 (0.65)	<0.0001
	Section B											
4	The degree of your comfort in talking about spirituality/religion	2.6	4	2.3	2.75	2.9125 (0.75)	4.4	4.6	4.3	4.33	4.41 (0.13)	<0.0001
5	The degree of your comfort in talking to patients	3	3.2	2.3	3.25	2.9375 (0.44)	5	3.8	4.3	4.67	4.44 (0.51)	<0.0001
6	The degree of your comfort in talking to patients about their spirituality or religious beliefs	2.8	2.2	1.1	2.25	2.0875 (0.71)	4.6	4.4	4	4.67	4.42 (0.3)	<0.0001

I think more providers than are willing to admit it are guided/motivated by spirituality or faith but worry about being looked down on by coworkers who think they are “beyond” religion.

How to better tap into the spirituality of my colleagues and see if I can help them access it, dig deeper, or be more fulfilled in whatever spirituality they want/need.

#### Comfort engaging patients around spirituality

Participants self-reported a significant increase in the degree of their comfort in talking about spirituality (*p* < 0.0001), the degree of their comfort in talking to patients (*p* < 0.0001), and the degree of their comfort in talking to patients about spirituality (*p* < 0.0001) at the end of the internship compared with its start (Table 6, Section B). Students rated their comfort in speaking to patients and in talking about spirituality, either generally or with patients, as fair to good at the start of the internship, but as very good to excellent at the end, indicating that the summer internship experience had helped to develop new skills and competencies in communicating with patients. Post-program responses corroborated students’ development, as participants’ responses to the question, *What were the three most important things you learned this summer*, included comments such as:

Learning concrete relational communication skills and strategies … were invaluable in exponentially increasing my ability to properly set up and gracefully navigate a conversation. These skills contribute greatly to my newfound confidence in engaging with patients.

I learned that is not awkward to ask patients, do you believe in God? Or, tell me, what supports you? That matters a lot to me, I previously had a misconception that such conversations could be awkward.

## Discussion

Over the past two decades, U.S. medical schools have increasingly added content on spirituality and spiritual care to their curricula, typically in the form of brief, *ad hoc*, elective experiences or in didactics and/or discussions embedded in required courses.^18–20^ In contrast, we describe the feasibility, design, content, and the first five years of outcomes for a more immersive, medical student summer internship based on an adaption of CPE that provides effective instruction on spirituality and health as well as fosters professional development. Given the need for novel approaches to medical student education around patient spirituality and spiritual care, we believe our findings represent an important contribution to this literature. Additionally, this pedagogical, interprofessional approach aligns with the efforts of the End-of-Life Nursing Education Consortium project^29^ and the Education in Palliative and End-of-Life Careprogram,^30^ and is responsive to the European Association of Palliative call for more multidisciplinary education for spiritual care in palliative care.^16^

Spiritual care and needs are routinely neglected in health care settings.^10–13^ As spiritual beliefs influence patients’ medical decision making, as well as impact the clinician-patient relationship,^1–3^ failing to attend to patient/family spirituality in the clinical relationship prevents physicians from providing culturally competent care. These deficiencies in the provision of spiritual care are due in part to inadequate training and provider discomfort.^18–20^ This situation persists because although 90% of U.S. medical schools now offer curricula in spiritual care,^18^ the vast majority of these offerings are isolated didactics/discussions or brief elective experiences, the content and quality of which vary widely, and which mostly occur during pre-clinical years when students often have fewer contacts with patients. Consequently, students have limited opportunities in their early training to directly apply this instruction to patient experiences. Considering these shortcomings, the SH-SIP in several ways constitutes a pedagogy that addresses the limitations of current approaches to spiritual care education for medical students.

First, the SH-SIP employs the framework of CPE, a validated pedagogy for training spiritual care professionals,^21–23^ with several features that lend themselves to medical student education in spirituality and health. CPE fosters self-awareness as a means of improving patient care.^24^ Studies have demonstrated that intensive weeks-long CPE training significantly increases emotional intelligence and self-reflection,^25^ suggesting that a six-week summer internship for medical students can also elicit meaningful change. And other health care fields, including programs for intensivists^26^ and health care administrators,^27^ have successfully adapted CPE, indicating a medical school-based adaptation is feasible. Consistent with this literature, participants in our internship rated the educational value and overall quality of the program highly and strongly endorsed they would recommend the SH-SIP to their peers (Table 4). We note, however, that while SH-SIP models its instruction after CPE, the goal is not to train students to be chaplains or spiritual care providers but to foster culturally competent care around spirituality.

Second, since the internship is offered during the summer, it circumvents the increasingly tight-for-time, inflexible pre-clinical U.S. medical school curricula, enabling a deeper experience for developing pertinent relational and professional skills.^31,32^ It is therefore not surprising that students highly valued the opportunity to visit patients (Table 5). By allowing students ample time to explore their own relationships with religion and spirituality, something likely to be difficult during the demanding school year, students are given meaningful opportunities to develop their self-awareness and skills of reflection. In line with this were our findings that participants reported significant increases in their awareness of how spirituality influences their life, their knowledge of the potential impact of spirituality on the patient experience, and their knowledge of the role of spirituality in the lives of health care providers (Table 6, Section A). Further, the extended time in conversation with patients enables students to intentionally develop their relational communication skills for eliciting both spiritual and nonspiritual concerns of patients as well as becoming more comfortable in these types of conversations. The benefits of these opportunities for unrushed conversations were reflected in the findings that participants reported significant increases in their comfort in talking to patients, talking about spirituality, and talking to patients about spirituality (Table 6, Section B).

Lastly, the SH-SIP centers on patient connection and relational conversation, rather than the clinically-focused, information-extracting conversations students are taught to do in their early training,^33,34^ and so enables an enhanced opportunity for students to be socialized toward seeking to genuinely know and understand patients. Consequently, the SH-SIP fosters student empathy in ways not readily attainable in the typical pre-clinical Doctoring curriculum.^35^ The development of this sense of humanism was reflected in the qualitative comments where students repeatedly wrote about themes of awe and privilege that came with hearing the stories of patients. Student comments such as *This internship gave me a great appreciation for the value of simply being with patients …* speak to the impact of the SH-SIP in fostering an “inner silence” that enables students to truly meet patient and family needs through presence.

Although potentially a novel approach for instructing medical students about spiritual care, we acknowledge the challenges of conducting a six-week, full-time, CPE-based summer internship like the SH-SIP. These include recruiting a certified CPE instructor capable of effectively engaging medical students; the costs associated with student stipends, administrative support, and compensation for the instructor; and the significant program logistics and coordination. Consequently, a strong institutional commitment is required for the success and sustainability of this type of educational initiative.

Several limitations are noted. First, as our study involved a small number of rising second-year medical students from a single institution, the generalizability of our findings remains to be determined. Second, since this was a summer internship, students self-selected into enrolling, potentially skewing for students who were more receptive to learning about religion and spirituality. Third, the data were self-reported, without objective assessments of learning. Lastly, data were only collected immediately after the completion of the program and so the durability of the impact of the SH-SIP is unknown. Consequently, future studies should involve other medical schools with larger numbers of students. Additionally, assessments should be included that use standardized patients and/or direct observation, and which are done immediately as well as at later time points after the internship.

Despite these limitations, the data presented demonstrate that a medical student summer internship focused on patient spirituality and spiritual care modeled after CPE provides a unique level of immersion not obtainable in the typical pre-clinical curriculum. Such a program is not only likely to be well received by students but is an effective approach for (1) improving students’ awareness of their own spirituality, and the role it can play in their professional careers; (2) enhancing student knowledge of issues pertinent to patient spirituality; and (3) fostering student empathy and their relational communication skills.

## Data Availability

The primary data for this study can be accessed at https://doi.org/10.17632/4mn5k3vjcj.1. The corresponding author can be contacted for requests for other information.

## Abbreviations Used

CPE: clinical pastoral education
SH-SIP: Spirituality and Health Summer Internship Program
