# Supplementary material for: Spirituality and Health Summer Internship Program: Adapting Clinical Pastoral Education for Medical Student Instruction in Patient Spirituality
Source: Palliat Med Rep. 2025 Apr 22;6(1):76–83. doi: 10.1089/pmr.2024.0101 (PMC12040561; doi:10.1089/pmr.2024.0101)
Supplement: Supplementary Data [file pmr.2024.0101_supplementary_data.pdf]

Spirituality and Health Summer Internship Program: Adapting Clinical Pastoral Education for Medical Student Instruction in Patient Spirituality

Supplemental Information

Pre-Program Survey Questions

Post-Program Survey Questions

Student Program Evaluation

Spirituality and Health Summer Internship Program
Perelman School of Medicine

Pre-Program Survey Questions

1. The degree of your awareness of your spiritualty and its impact or influence on your life

**1**	**2**	**3**	**4**	**5**
Poor	Fair	Good	Very Good	Excellent

2. The degree of your knowledge of the potential impact or influence of spirituality on the patient experience

**1**	**2**	**3**	**4**	**5**
Poor	Fair	Good	Very Good	Excellent

3. The degree of your knowledge of the potential role of spirituality in the lives (personal and professional) of healthcare providers

**1**	**2**	**3**	**4**	**5**
Poor	Fair	Good	Very Good	Excellent

4. The degree of your comfort in talking about spirituality/religion

**1**	**2**	**3**	**4**	**5**
Poor	Fair	Good	Very Good	Excellent

5. The degree of your comfort in talking to patients

**1**	**2**	**3**	**4**	**5**
Poor	Fair	Good	Very Good	Excellent

6. The degree of your comfort in talking to patients about their spirituality or religious beliefs

**1**	**2**	**3**	**4**	**5**
Poor	Fair	Good	Very Good	Excellent

7. List three things you hope to learn during the Spirituality and Health Summer Internship Program

Spirituality and Health Summer Internship Program
Perelman School of Medicine

Post-Program Survey Questions

1. The degree of your awareness of your spiritualty and its impact or influence on your life

**1**	**2**	**3**	**4**	**5**
Poor	Fair	Good	Very Good	Excellent

2. The degree of your knowledge of the potential impact or influence of spirituality on the patient experience

**1**	**2**	**3**	**4**	**5**
Poor	Fair	Good	Very Good	Excellent

3. The degree of your knowledge of the potential role of spirituality in the lives (personal and professional) of healthcare providers

**1**	**2**	**3**	**4**	**5**
Poor	Fair	Good	Very Good	Excellent

4. The degree of your comfort in talking about spirituality/religion

**1**	**2**	**3**	**4**	**5**
Poor	Fair	Good	Very Good	Excellent

5. The degree of your comfort in talking to patients

**1**	**2**	**3**	**4**	**5**
Poor	Fair	Good	Very Good	Excellent

6. The degree of your comfort in talking to patients about their spirituality or religious beliefs

**1**	**2**	**3**	**4**	**5**
Poor	Fair	Good	Very Good	Excellent

7. List the three most things you learned during the Spirituality and Health Summer Internship Program

Spirituality and Health Summer Internship Program
Student Program Evaluation

GENERAL QUESTIONS

1. **Clarity of program goals, objectives and expectations**

   O 1 (poor) O 2 (Fair) O 3 (Good) O 4 (Very good) O 5 (Excellent)

2. **How well the program achieved stated goals**

   O 1 (poor) O 2 (Fair) O 3 (Good) O 4 (Very good) O 5 (Excellent)

3. **Educational value of the program**

   O 1 (poor) O 2 (Fair) O 3 (Good) O 4 (Very good) O 5 (Excellent)

4. **Overall rating/quality of program**

   O 1 (poor) O 2 (Fair) O 3 (Good) O 4 (Very good) O 5 (Excellent)

5. **I would recommend this program to other medical students**

   O 1 (Disagree strongly) O 2 (Disagree) O 3 (Neutral) O 4 (Agree) O 5 (Agree Strongly)

PROGRAM ACTIVITIES

Please rate the following activities

1. **Patient visitations**

   O 1 (poor) O 2 (Fair) O 3 (Good) O 4 (Very good) O 5 (Excellent)

2. **Mindfulness meditation training**

   O 1 (poor) O 2 (Fair) O 3 (Good) O 4 (Very good) O 5 (Excellent)

3. **Visitation presentation**

   O 1 (poor) O 2 (Fair) O 3 (Good) O 4 (Very good) O 5 (Excellent)

4. **Individual sessions**

   O 1 (poor) O 2 (Fair) O 3 (Good) O 4 (Very good) O 5 (Excellent)

5. **Interpersonal group conversations**

   O 1 (poor) O 2 (Fair) O 3 (Good) O 4 (Very good) O 5 (Excellent)

6. **Individual narrative (life stories) presentations**

   O 1 (poor) O 2 (Fair) O 3 (Good) O 4 (Very good) O 5 (Excellent)

7. **Physician Faculty Interview**

   O 1 (poor) O 2 (Fair) O 3 (Good) O 4 (Very good) O 5 (Excellent)

8. **Capstone presentations**

   O 1 (poor) O 2 (Fair) O 3 (Good) O 4 (Very good) O 5 (Excellent)

PLEASE PROVIDE ANY OTHER COMMENTS OR SUGGESTIONS FOR IMPROVEMENT
